# One-step Preparation of Nanoarchitectured TiO_2_ on Porous Al as Integrated Anode for High-performance Lithium-ion Batteries

**DOI:** 10.1038/srep20138

**Published:** 2016-02-04

**Authors:** Xianfeng Du, Qianwen Wang, Tianyu Feng, Xizi Chen, Liang Li, Long Li, Xiangfei Meng, Lilong Xiong, Xiaofei Sun, Lu Lu, Youlong Xu

**Affiliations:** 1 Electronic Materials Research Laboratory, Key laboratory of the Ministry of Education & International Center of Dielectric Research, Xi’an Jiaotong University, Xi’an, 710049, China

## Abstract

Titanium dioxide (TiO_2_) is an attractive anode material for energy storage devices due to its low-volume-change and high safety. However, TiO_2_ anodes usually suffer from poor electrical and ionic conductivity, thus causing dramatic degradation of electrochemical performance at rapid charge/discharge rates, which has hindered its use in energy storage devices. Here, we present a novel strategy to address this main obstacle via using nanoarchitectured TiO_2_ anode consisting of mesoporous TiO_2_ wrapped in carbon on a tunnel-like etched aluminum substrate prepared by a simple one-step approach. As a result of this nanoarchitecture arrangement, the anode exhibits excellent rate performance and superior cyclability. A rate up to 100 C is achieved with a high specific capacity of about 95 mA h g^−1^, and without apparent decay after 8,000 cycles.

Lithium-ion batteries (LIBs) have been widely applied in portable electronic devices due to their superior properties such as high energy density, light weight and low toxicity^1^,^2^,^3^,^4^,^5^,^6^. Titanium dioxide (TiO_2_), as a promising high-performance anode for LIBs, has attracted increasing attention in recent years because of its low volume expansion, low cost as well as non-toxicity^7^,^8^,^9^,^10^,^11^. Moreover, the relatively high lithium insertion/extraction voltage (higher than 1.5 V vs. Li/Li^+^) can efficiently avoid the formation of SEI layers and dendritic lithium, improving the batteries safety compared to its carbon counterparts^1^,^8^,^9^,^12^,^13^. However, low electrical conductivity (ca. 10^−12^–10^−7^ S cm^−1^) and Li^+^ diffusivity (ca. 10^−15^–10^−9^ cm^2^ s^−1^) hinder its practical application in high-power LIBs^12^,^14^,^15^,^16^,^17^,^18^. To address these issues, shortening the characteristic dimensions of TiO_2_ or/and coating TiO_2_ with conductive layer have been widely considered as efficient approaches for enhancing ion and electron transport kinetics in batteries. And various strategies have been developed so far, such as exploiting TiO_2_ with a variety of nanostructures (e.g., nanosphere^19^, nanowire^20^, nanotube^21^, nanoribbon^22^, etc.), preparing hybrid with carbon-based materials (e.g., TiO_2_/C^23^, TiO_2_/CNTs^24^, TiO_2_/graphene^12^, TiO_2_/fullerene^25^, etc.). Unfortunately, the application of these nanoscaled TiO_2_ to specific areas always suffers from low volumetric energy density, poor inter-particle contacts and self-aggregation upon deep cycling. In this regard^26^, mesoporous TiO_2_ is proposed to offer a clever solution to achieve high volumetric energy densities for LIBs. Nevertheless, only modest improvements in rate performance have been observed, because the mesoporous structures primarily address ion but not electron transport^27^. More efforts, therefore, are urgently needed to develop integrated electrode including active materials and architecture substrate.

A perfect electrode with excellent rate performance and high capacity requires the simultaneous minimization of five resistances present during charging/discharging (Fig. 1c): (1) ion transport in the electrolyte, (2) ion transport in the electrode, (3) electrochemical reactions in the electrode, (4) electron transport in the electrode and (5) electron conduction in the current collector^27^. Comparing with conventional electrodes (Fig. 1a), nanoarchitectured electrode with a three-dimensional interpenetrating network of hybrid nanoscaled active materials and nano- or micro- structured current collectors seems to be a promising avenue to meet these requirements, as stated below^27^,^28^,^29^,^30^. First, hybrid nanoscaled active materials can evidently shorten the ion diffusion length and offer tremendous active surface areas, which benefits (2) and (3). Second, nano- or micro- structured current collectors are capable of providing efficient pathways for ion and electron transport through the entire electrode architecture, which is conductive to (1), (4) and (5). In addition, nano- or micro- structured current collectors have much larger surface areas and less mass than the planar bulky ones do, which dramatically increases the loading of active materials and decreases the total mass of the electrode, thus improving the volumetric and gravimetric energy density. Since the pioneering work by Taberna in 2006^31^, the fabrication of these structures, such as coaxial nanoarrays^32^, metal foam^33^, porous metals^34^, conducting polymer scaffolds^35^ and inverse opal matrix^27^, has drawn worldwide interest. In most of the previous reports, however, the fabrication usually required somewhat complex chemical and physical processes and cost a lot, which is not in favor of the large-scale manufacture.

Here, we present a novel strategy for the fabrication of nanoarchitectured electrode consisting of mesoporous TiO_2_ wrapped by carbon on a tunnel-like etched aluminum foil (Fig. 1b) via an inexpensive one-step approach. The etched aluminum foil is chosen as the structure support for the electroactive TiO_2_ because of its large surface area (0.3–1.5 m^2^ g^−1^), high conductivity (1–3 × 10^7^ S m^−1^), excellent mechanical flexibility (tensile strength: 20–30 MPa; bending strength: 40–80 round cm^−1^), low cost (15–25 $ Kg^−1^) and commercial availability. The preparation process is schematically illustrated in Fig. 1d. To start with, a TiO_2_ precursor was prepared via mixing tetrabutyl titanate and isopropyl alcohol. Trace of hydrogen fluoride was also added into the precursor to eliminate the oxide on aluminum foil and improve electrical contact of TiO_2_ with support. The etched aluminum foils were then dipped into the obtained precursor for anchoring TiO_2_. After calcined in atmosphere at temperature of 400, 450, 500 °C, respectively, for 10 mins, a nanoarchitectured TiO_2_ electrode was obtained (denoted as TO/Al-400, TO/Al-450, TO/Al-500 or TO/Al hereafter). Finally these electrodes were directly used as binder-free anodes in LIBs.

## Results and Discussion

The field-emission scanning electron microscope (FE-SEM) images of etched Al substrate are shown in Fig. 2a,b and Figure S1 (see the supporting information). It is observed that the etched Al substrate is porous framework made of tunnel-like hole with uniform diameter of about 1 μm and length of tens of micrometers. These holes perpendicular to the surface benefits the transport of electrolyte. After dip-coating and calcination, the Al substrates are covered by a layer of TiO_2_. FE-SEM examination (Fig. 2d,e,g,h,j,k) shows that TiO_2_ are uniformly anchored onto the Al substrate to form a dense layer with the thickness of about 100 nm. The thickness changes few with the calcination temperature, which agrees well with the tiny weight loss in Figure S2b. Energy-dispersive X-ray spectroscopy (EDX) measurement indicates the presence of Ti, O, C and Al elements after dip-coating (see Fig. 2f,i,l). There is no F peak observed because of the trace amount of fluorine. However, a very weak peak of F 1s can be observed for TO/Al-400, TO/Al-450 and TO/Al-500 samples via X-ray photoelectron spectroscopy (XPS), which has higher detection sensitivity (as Figure S3(a)).

Grazing incidence X-ray diffraction (GIXRD) analysis in Fig. 3a shows that after annealing, the deposited TiO_2_ converts into crystal TiO_2_ identified as anatase (JCPDS card No. 01-071-1167), as characterized by the appearance of peaks from (101), (004), (200), (105), (211), (204), (116), (220) and (215). And these peaks become intensive and sharp with increasing annealing temperature, which indicates the crystallinity of TiO_2_ is improved. The Raman spectra in the range of 30–800 cm^−1^ (see Fig. 3b) show the normal modes of anatase at 166, 212, 405, 522 and 643 cm^−1^, assigned to E_g_, E_g_, B_1g_, A_1g_ and E_g_ modes, respectively^36^,^37^. The intensity of E_g_(1) increases with increasing annealing temperature, indicating the crystallinity becomes better, which corresponds to the result of GIXRD. The Raman spectra in the range of 1000–1800 cm^−1^ are also collected to detect the residual carbon in TiO_2_ film and presented in Fig. 3c. There exist two bands at ~1380 cm^−1^ and ~1590 cm^−1^, named D band and G band, respectively. The D band corresponds to disordered carbon or defective graphitic structure, while the G band, identified as the tangential vibration of carbon atoms, is a characteristic feature of graphitic layers^38^,^39^. It is shown, from Fig. 3c, that these samples contain the carbon with partial graphitization, which is highly desirable for the application as electrode material due to its high conductivity. The amount of this kind of carbon varies with the annealing temperature. Before 500 °C, the intensity of D and G band is high, meaning a lot of carbon remained in samples. However, the intensity of these two bands decreases dramatically when annealed at 500 °C, indicating the amount of residuary carbon drops sharply. This is caused by combustion of carbon as shown in Figure S2c. The optical images of these samples (Fig. 3d) display that the color of TO/Al-400 and TO/Al-450 is darker than that of TO/Al-500, which suggests that more carbon exists in TO/Al-400 and TO/Al-450 comparing to TO/Al-500. The content of carbon in these samples is calculated via Equation S1-S4. The results reveal that the percentage of carbon in TO/Al-400, TO/Al-450 and TO/Al-500 is 50–53%, 44–49% and 21–28%, respectively. Here it is important to note that in all samples Ti element exists as the form of TiO_2_ because there is only + 4 Ti in TO/Al samples demonstrated by XPS measurement, as shown in Figure S3(b).

Closer observations by FE-SEM and transmission electron microscope (TEM) on morphology and structure of the anchored TiO_2_ film are given in Fig. 4 and Figure S4. A high-magnification SEM image (Fig. 4a) exhibits the TiO_2_ film is porous, which is further manifested by TEM measurements as shown in Fig. 4b. High angle annular dark field-scanning transmission electron microscope (HAADF-STEM) image (Fig. 4c and Figure S4) discovers typical mesoporous characteristics with a narrow distribution in range of 1–10 nm in the TiO_2_ film, which is consistent with the result of Brunner-Emmet-Teller (BET) measurement (Figure S5). EDX mapping (Fig. 4d–g) detects a large amount of carbon distributed within the TiO_2_ film. High-resolution TEM (HRTEM) (Fig. 4h–j) analysis reveals TiO_2_ film possesses a nanonetwork in which TiO_2_ nanocrystals are wrapped by carbon. With the increase in annealing temperature, the amount of carbon decreases and more anatase TiO_2_ appears. The lattice fringes with a distance of 0.237 nm are observed in Fig. 4h–j, corresponding to the (004) interplane spacing of anatase.

To evaluate the LIBs applications of these unique TO/Al binder-free electrodes, the electrochemical performance was investigated. Figure 5a shows the cyclic voltammograms (CV) of the TO/Al electrodes annealed at different temperature at a slow scan rate of 0.1 mV s^−1^. A pair of cathodic/anodic peaks centered at about 1.72/2.02 V, corresponding to the lithium insertion/extraction in anatase lattice, can be observed. The gap between anode and cathode peaks is 0.30, 0.30 and 0.39 V for TO/Al-400, TO/Al-450 and TO/Al-500, respectively, indicating weaker polarization in TO/Al-400 and TO/Al-450 than that in TO/Al-500. Electrochemical impedance spectra (EIS) show the charge transfer resistance increases in order of TO/Al-450, TO/Al-400, and TO/Al-500 (see Figure S6 and Table S1). This attributes to the different amount of carbon and various crystallinity of TiO_2_. The CV curves at different scan rate indicates that the peak current density perfectly scales with the square root of scan rate (Figure S7), evidencing a semi-infinite diffusion process in the TiO_2_ film at high current rate. According to Randles-Sevick equation^13^,^40^, the Li^+^ apparent chemical diffusion coefficient increases in order of TO/Al-400 (1.47 × 10^−13^ cm^2^ s^−1^) <TO/Al-500 (2.61 × 10^−13^ cm^2^ s^−1^) <TO/Al-450 (4.45 × 10^−13^ cm^2^ s^−1^). Figure 5b presents the initial discharge-charge voltage profiles at a current density of 0.3 C (where 1C rate represents one-hour completely charge or discharge the practical capacity determined experimentally) within a cut-off voltage window of 1.0-3.0 V. The initial discharge/charge capacities are found to be 730/328, 632/335 and 473/270 mA h g^−1^ (based on the mass of TiO_2_) for TO/Al-400, TO/Al-450 and TO/Al-500, respectively. The irreversible capacity in the first cycle is mainly due to the interfacial reaction between TiO_2_ and the electrolyte, which is common in most lithium intercalation hosts^7^,^41^. As annealing temperature rises, the coulombic efficiency is improved, which maybe owns to the loss of H_2_O and O-H groups on the surface of TiO_2_ caused by the heat (see Figure S8 and Table S2). After couple cycles, nonetheless, the coulombic efficiency can be raised to nearly 100%, as shown in Figure S9a. From the second cycle onwards, the TiO_2_ electrodes show good reversibility. And at the end of 200 discharge-charge cycles, a reversible capacity of 317 (TO/Al-400), 316 (TO/Al-450) and 233 (TO/Al-500) mA h g^−1^ can be retained. In present work, the electrochemical performance of blank Al substrate and pyrolytic carbon on Al substrate were also measured and the results are shown in Fig. 5a, Figure S10 and S11. Within operating voltage window of 1.0–3.0 V, the capacity contributed by Al (<1 mA h g^−1^) or pyrolytic carbon (<12 mA h g^−1^) is negligible because the lithium intercalation within Al or carbon mainly occurs at potential far below 1.0 V^42^,^43^.

Benefiting from the unique structure, the TiO_2_ electrode exhibits excellent rate performance, as shown in Fig. 5c. The results reveal that TO/Al-400 and TO/Al-450 present better reversibility and higher capacity than that of TO/Al-500 at different current rates. This mainly profits from the high amount of carbon. Compared with TO/Al-400, TO/Al-450 shows more dominant performance especially at high discharge/charge rates. For example, TO/Al-450 electrode delivers charge capacity of 153 mA g^−1^ at 20 C, 117 mA g^−1^ at 50 C, and 95 mA g^−1^ at 100 C, whereas the values for TO/Al-400 are 141 mA g^−1^, 99 mA g^−1^, and 78 mA g^−1^, respectively. This is possibly caused by the better crystallinity of TO/Al-450. Importantly, after more than forty cycles tested under various current rates even up to 100 C, the capacities of TO/Al electrodes can recover to the initial value at different current rates, indicating its high reversibility of lithium ion insertion/extraction in the electrode. The specific energy density and specific power density calculated from discharge profiles are also displayed as the Ragone plot as shown in Figure S12. Apparently, the maximum specific energy density of 141 Wh kg^−1^ is achieved for our TO/Al-450 sample at highest specific power density of 14.1 kW kg^−1^. Further, long cycling performance at high current rates of 20, 50, and 100 C are also investigated and shown in Fig. 5d and Figure S9b,c. The TO/Al electrodes exhibits excellent stability and superior cyclability and it, even after 8000 cycles at 100 C, still affords a remarkable capacity of 97 mA h g^−1^ for TO/Al-450, which is better than TO/Al-500 (60 mA h g^−1^) and TO/Al-400 (81 mA h g^−1^). This can be explained that TO/Al-450 has high amount of carbon as well as good crystallinity. These exciting performances are better than most of the reports on nano-TiO_2_.

Clearly, the exceptional electrochemical performance of TO/Al electrode is originated from the unique structure merits. First, mesoporous TiO_2_ offers infinite open channels to facilitate the Li^+^ ion transport and huge surface area (The surface areas of TiO_2_ and carbon deposited on Al substrate after calcined at 400, 450, and 500 °C is about 85, 81, and 67 m^2^ g^−1^, respectively. see Figure S5) to favor the Li^+^ ion insertion/extraction. Second, the ultrathin mesoporous structure greatly shortens the ionic diffusion length and improves the architecture stability. Third, the etched Al substrate has numerous tunnel-like holes which provides efficient pathways for electrolyte transport through the whole electrode and evidently shortens the electronic transport distance. Fourth, the 3D network of TiO_2_ wrapped by carbon not only enhances the electronic conductivity of the electrode, but also restrains the aggregation of titanium clusters and maintains the nanostructure during cycles. The last, the introduction of trace HF maybe give rise to a better electrical contact and lower internal resistances via cleaning the Al surface, resulting in an excellent rate performance (Figure S13).

## Conclusions

In summary, we have presented a simple strategy to fabricate high-rate rechargeable anodes. As-prepared 3D nanoarchitectured electrode provides numerous pathways for ions and electrons transport as well as extremely shortened ionic/electronic diffusion length. Moreover, the processes do not involve any complex equipment and are entirely compatible with many battery chemistries. The approach may even enable the use of those materials with low ion and electron conductivities because of the short diffusion length provided by nanoarchitectured electrode. However, further research is needed to increase the loading mass of active material and to reduce the content of carbon in active material, promoting the application of this approach to practical batteries. A viable route to this end could be to adjust the diameter and length of pore in Al substrate and to use Ti precursor with less carbon content. We believe that the nanoarchitecture concept we have described may hold great promise for the development of LIBs with high power and energy densities and can be extended as a general approach to other systems such as supercapacitors.

## Methods

### Material

All chemicals were of analytical grade and used as received without further purification. Tetrabutyl titanate (TBT), isopropanol, sodium hydroxide, hydrochloric acid, and hydrofluoric acid were purchased from Sinopharm Chemical Reagent Co., Ltd., China. Deionized water was prepared by Milli-Q-Reference water system (Millipore Co., USA). High purity (99.99%) commercially etched aluminum foils with tunnel-like pores (110 μm, 630 V/0.61 μF cm^−2^, Zhaoqing Huafeng Co. Ltd, China) were used as substrates.

### Electrode Preparation

**Figure 1:**
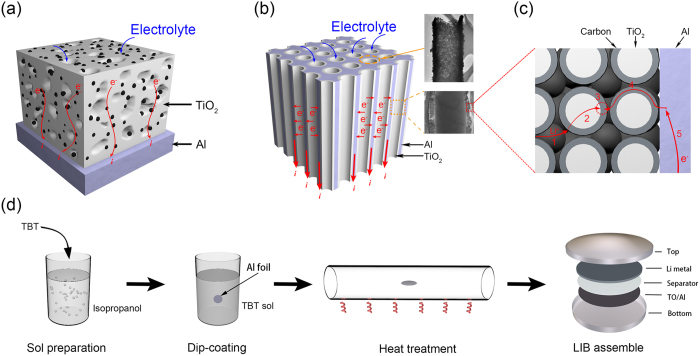
Schematic of traditional electrode (**a**) and novel electrode in present work (**b**). Illustration of the five primary resistances in a battery electrode (**c**). Flow chart for TO/Al electrode preparation and LIBs assembly (**d**). The insets in (**b**) are TEM and SEM images of TiO_2_ deposited on Al substrate.

**Figure 2:**
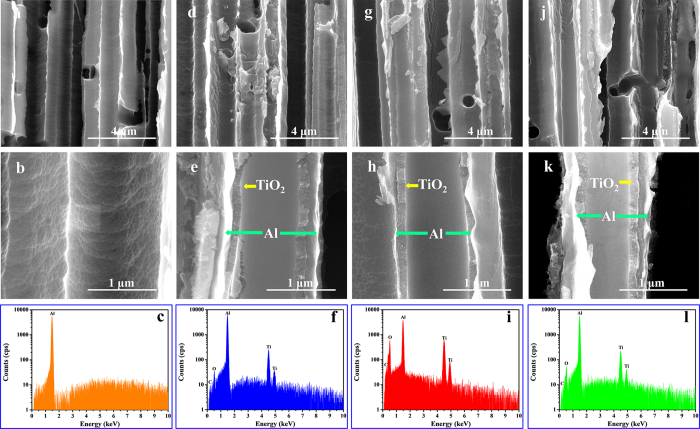
FE-SEM images and EDAX patterns of blank Al substrate (**a**–**c**), TO/Al-400 (**d**–**f**), TO/Al-450 (**g**–**i**), and TO/Al-500 (**j**–**l**).

**Figure 3:**
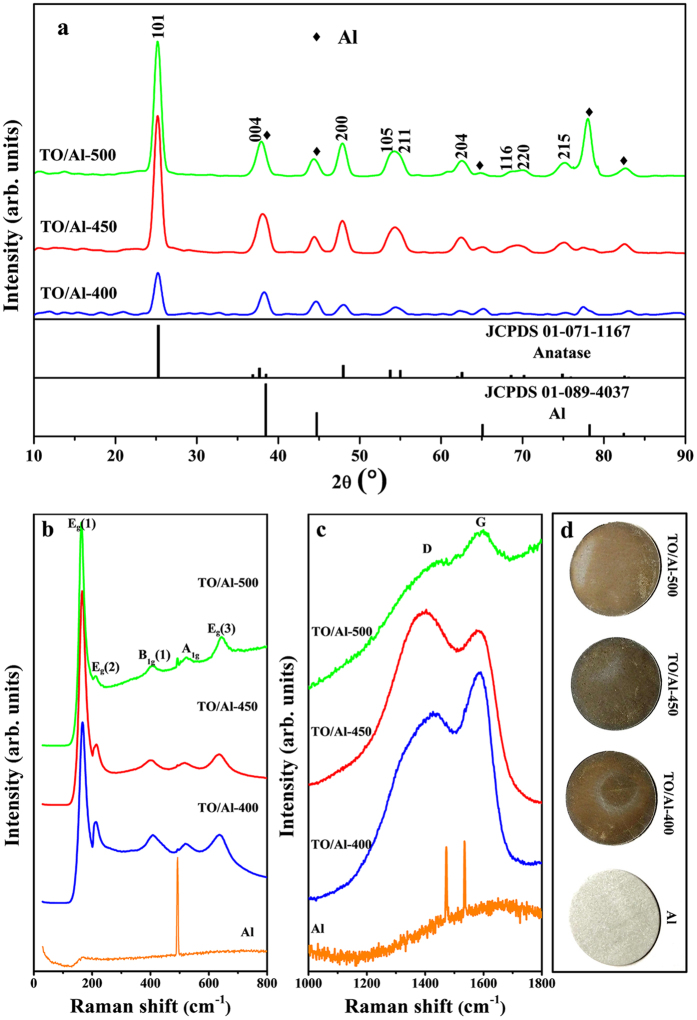
(**a**) GIXRD patterns of TO/Al-400, TO/Al-450, TO/Al-500 along with reference patterns of TiO_2_ anatase phase (JCPDS 01-071-1167) and Al (JCPDS 01-089-4037); (**b**,**c**) Raman spectra of blank Al substrate, TO/Al-400, TO/Al-450, and TO/Al-500 in the range of 0-800 cm^−1^ and 1000-1800 cm^−1^, respectively; (**d**) optical images of blank Al substrate, TO/Al-400, TO/Al-450, and TO/Al-500.

**Figure 4:**
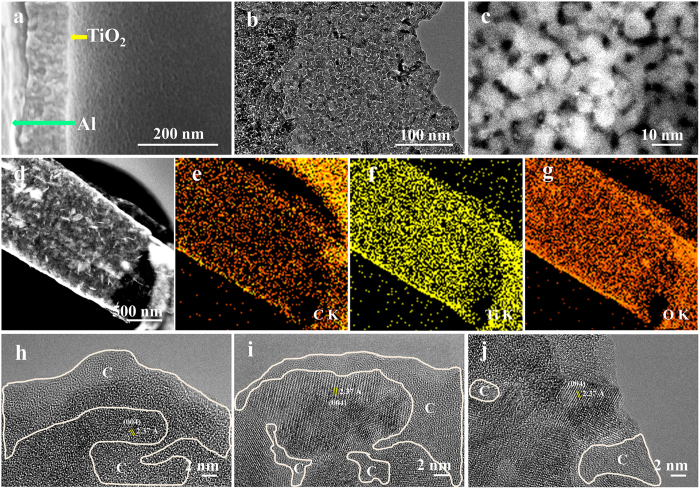
(**a**) FE-SEM, (**b**) TEM, (**c**) HAADF-STEM, (**d**–**g**) EDX mapping images of TO/Al-450 and HRTEM images of TO/Al-400, TO/Al-450, TO/Al-500.

**Figure 5:**
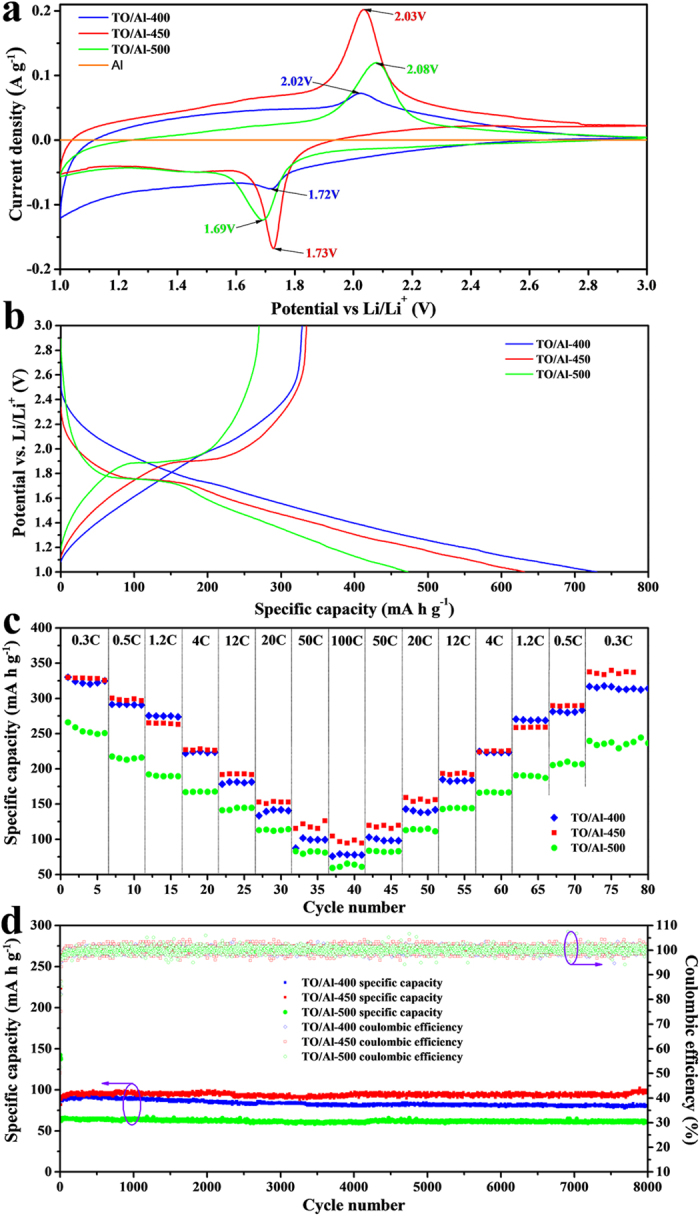
Electrochemical performance of TO/Al-400, TO/Al-450, and TO/Al-500 in the voltage window of 1–3 V. (**a**) CV curves of blank Al substrate, TO/Al-400, TO/Al-450, and TO/Al-500 at scan rate of 0.1 mV s^−1^; (**b**) initial charge/discharge curves at a current rate of 0.3 C; (**c**), rate capability tests from 0.3 to 100 C; (**d**) cycle performance at a current rate of 100 C after 0.3 C for the first 3 cycles.

The binder-free electrodes were prepared by dip-coating using TiO_2_ precursor, followed by heat-treatment in air, as illustrated in Fig. 1d. Typically, TBT was firstly dissolved in isopropanol solution containing 2.5 mmol L^−1^ hydrogen fluoride to form a homogenous precursor with a molar ratio of 500 mmol L^−1^. Then, tunnel-like Al substrates were conducted to dip into the above solution for deposition of TiO_2_. After which, the resultant samples were rapidly pushed into tube furnace at desired temperature (400, 450 and 500 °C, respectively). After 10 min, they were pulled out immediately and put in atmosphere, a nanoarchitectured TiO_2_ electrodes were obtained. These electrodes were directly used as binder-free anodes in LIBs.

### Material Characterization

The morphology and composition of samples were investigated with field-emission scanning electron microscope (FE-SEM, Quanta 250FEG, FEI, USA) and TEM (JEM-2100, JEOL, Japan). In order to detect the morphology of TiO_2_ deposited on the surface of Al by TEM, TO/Al electrodes were first eroded with 5 mol·L^−1^ sodium hydroxide to obtain TiO_2_ powder as shown in Figure S14. The crystalline structure of TO/Al electrodes was characterized by X-ray diffraction (XRD, X’Pert PRO, PANalytical, Holland) and Raman spectroscopy (Jobin Yvon LabRAM HR800, ENS, Lyon, France). TiO_2_ evolution on Al substrate during heat-treatment process was investigated with thermogravimetry-differential scanning calorimetry (TG-DSC, TGA/DSC 1, METTLER TOLEDO, Switzerland). The OH group on the surface of TiO_2_ was detected by Fourier transforms infrared (FTIR, Avatar 360 FTIR ESP, Thermo Nicolet, USA). The elemental chemical state of the samples was examined via X-ray photoelectron spectroscopy (XPS, AXIS ULTRABLD, Kratos, Japan). The surface area and pore-size distribution were measured by Brunner-Emmet-Teller (BET, ASAP 2020, Micromeritics, USA). The content of titanium element in TO/Al samples was detected by an inductively coupled plasma-optical emission spectrometry (ICP-OES, 710, Agilent, USA) after dissolving TO/Al sample in the solution containing 1 mol L^−1^ hydrochloric acid and 0.1 mol L^−1^ hydrofluoric acid. The mass of samples was weighed by high-precision analytical balance (XS105DU, METTLER TOLEDO, Switzerland).

### Electrochemical Measurements

For the galvanostatic charging-discharging tests, a CR2016 coin-type cell was assembled in argon-filled glove box (Super(1225/750), MIKROUNA, China). In which Al foil loaded with TiO_2_ was directly used as working electrodes, lithium foil was used as the counter and reference electrodes, and a solution of 1.0 M LiPF_6_ in ethylene carbonate: diethyl carbonate (EC:DEC = 1:1 by weight) was used as the electrolyte. All the tests were conducted on a battery tester (CT2001A, LAND, China; BT2000, Arbin, USA) within 1.0–3.0 V at various current rates. The mass of TiO_2_ loaded was 0.7–1.0 mg cm^−2^ (the mass ratio between TiO_2_ and the Al current collector was 1:30-1:20). The specific capacities are calculated based on the mass of TiO_2_ in TO/Al samples. The electrochemical impedance spectroscopy (EIS) and cyclic voltammetry (CV) study were carried out on an electrochemical workstation (VMP2, Princeton, USA) in a three-configuration, with TO/Al as the working electrode and Li foil as both reference and counter-electrodes.

## Additional Information

**How to cite this article**: Xianfeng, D. *et al*. One-step Preparation of Nanoarchitectured TiO_2_ on Porous Al as Integrated Anode for High-performance Lithium-ion Batteries. *Sci. Rep*. **6**, 20138; doi: 10.1038/srep20138 (2016).

## Supplementary Material

Supplementary Information
