# Supplementary material for: One-step Preparation of Nanoarchitectured TiO2 on Porous Al as Integrated Anode for High-performance Lithium-ion Batteries
Source: Sci Rep. 2016 Feb 4;6:20138. doi: 10.1038/srep20138 (PMC4740746; doi:10.1038/srep20138)
Supplement: Supplementary Information [file srep20138-s1.pdf]

Supplementary Information

One-step Preparation of Nanoarchitectured TiO_2 on Porous Al as Integrated Anode for High-performance Lithium-ion Batteries

*Xianfeng Du, Qianwen Wang, Tianyu Feng, Xizi Chen, Liang Li, Long Li, Xiangfei Meng, Lilong Xiong, Xiaofei Sun, Lu Lu, and Youlong Xu**

*Email: ylxu@mail.xjtu.edu.cn

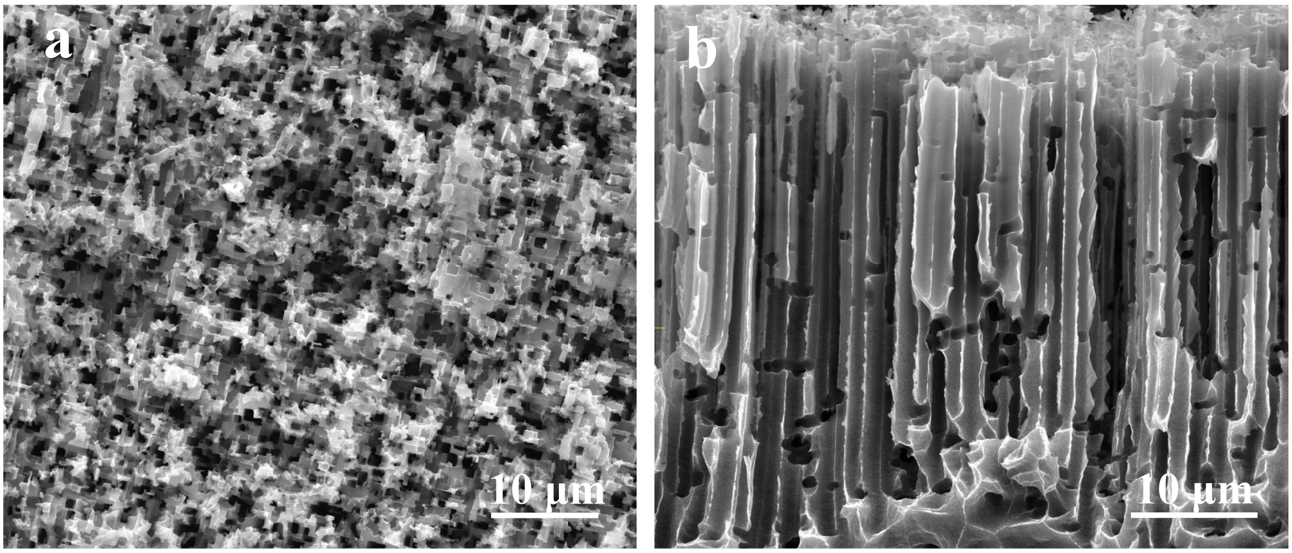

Figure S1. FE-SEM images of blank Al substrate: (**a**) surface and (**b**) cross-section.

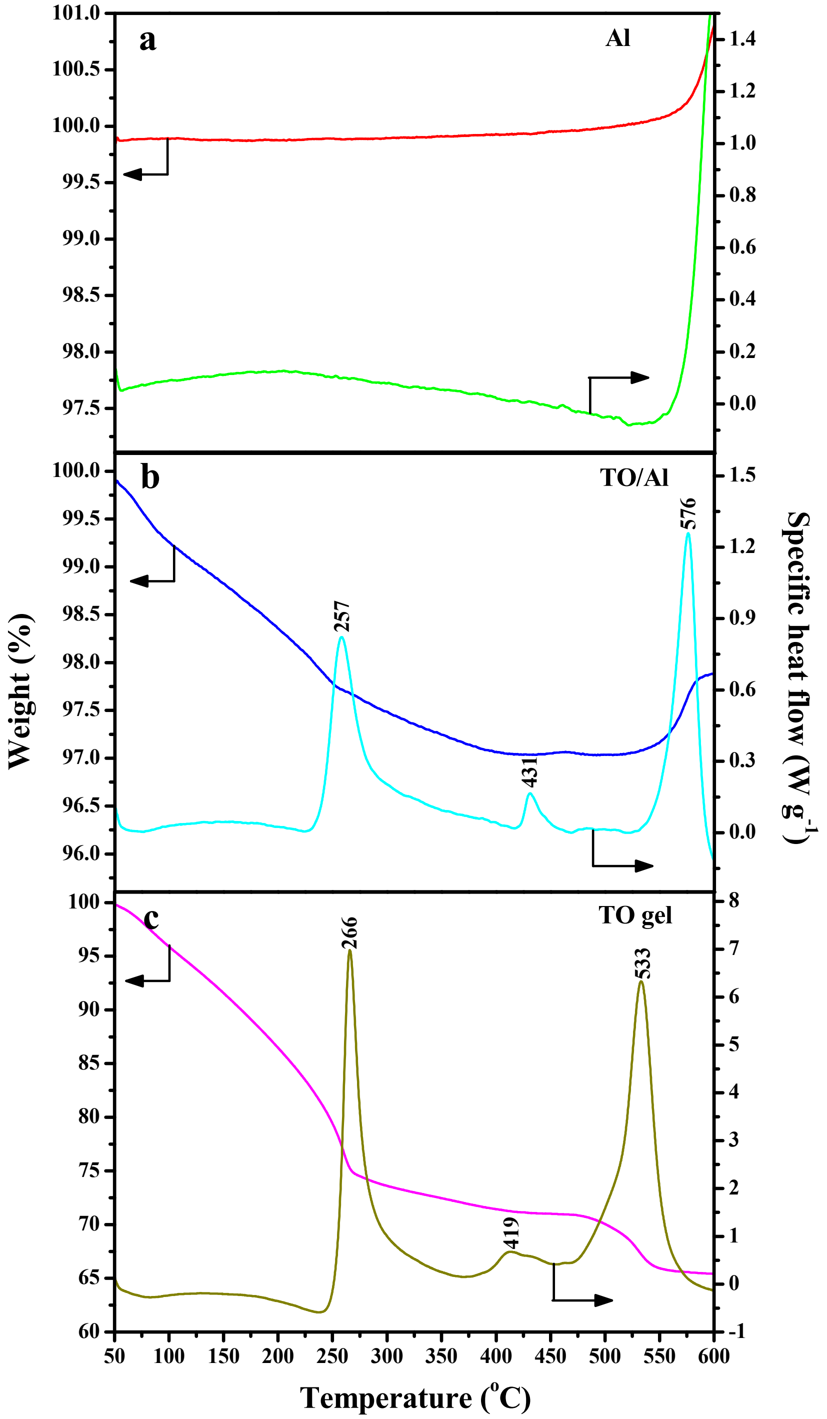

Figure S2. TG-DSC curves of Al (**a**) , TO/Al (**b**) , and TO gel (**c**) measured from 50 to 600 °C in air flow with a heating rate of 10 °C min^{-1}.

The as-deposited TiO_2 film is amorphous and contains a huge amount of organic groups and residues. Therefore, a thermal treatment is employed to remove these organic matters and improve

the crystallinity. The heat-treatment process is investigated via thermogravimetry-differential scanning calorimetry (TG-DSC) and the results are shown in **Figure S2**. For the blank Al substrate (**Figure S2a**), its weight hardly changes until 550 ^{o}C, after which, caused by the oxidation of Al, the weight increases sharply and a vast heat releases. While, for the TiO_2 gel, there are one endothermic and three exothermic peaks located at 50-110, 240-350, 375-450 and 475-600 ^{o}C, respectively. The endothermic peak corresponds to the evaporation of organics. The first exothermic peak is caused by the combustion and pyrolysis of organics. The second exothermic peak may be due to the crystallization of TiO_2. And the last exothermic peak attributes to the combustion of pyrolitic carbon. When TiO_2 precursor is covered on Al substrate, the evolution of TiO_2 during the annealing process (**Figure S2b**) is similar to that of TiO_2 gel (**Figure S2c**).

$$\Delta m = m_{TO/Al} - m_{Al}$$ **Equation S1**

$$m_{TiO_2} = \frac{m_{Ti}}{M_{Ti}/M_{TiO_2}}$$ **Equation S2**

$$C_{TiO_2} = \frac{m_{TiO_2}}{\Delta m} \times 100\%$$ **Equation S3**

$$C_{carbon} = 1 - C_{TiO_2}$$ **Equation S4**

where Δm is the total mass of TiO_2 and carbon deposited on Al substrate (The mass of Al_2O_3 caused by thermal oxidation is ignorable according to the TG-DSC test as shown in **Figure S2a**); $m_{TO/Al}$ and m_{Al} are the mass of TO/Al sample annealed at different temperature and Al substrate; m_{TiO_2} is the mass of TiO_2 in TO/Al samples; m_{Ti} is the mass of Ti element in TO/Al samples; M_{Ti} and M_{TiO_2} are the molecular weight of Ti and TiO_2; C_{TiO_2} and C_{carbon} are the content of TiO_2 and carbon in TO/Al samples. $m_{TO/Al}$ and m_{Al} were weighted by high-precision analytical balance. m_{Ti} was measured by an inductively coupled plasma-optical emission spectrometry.

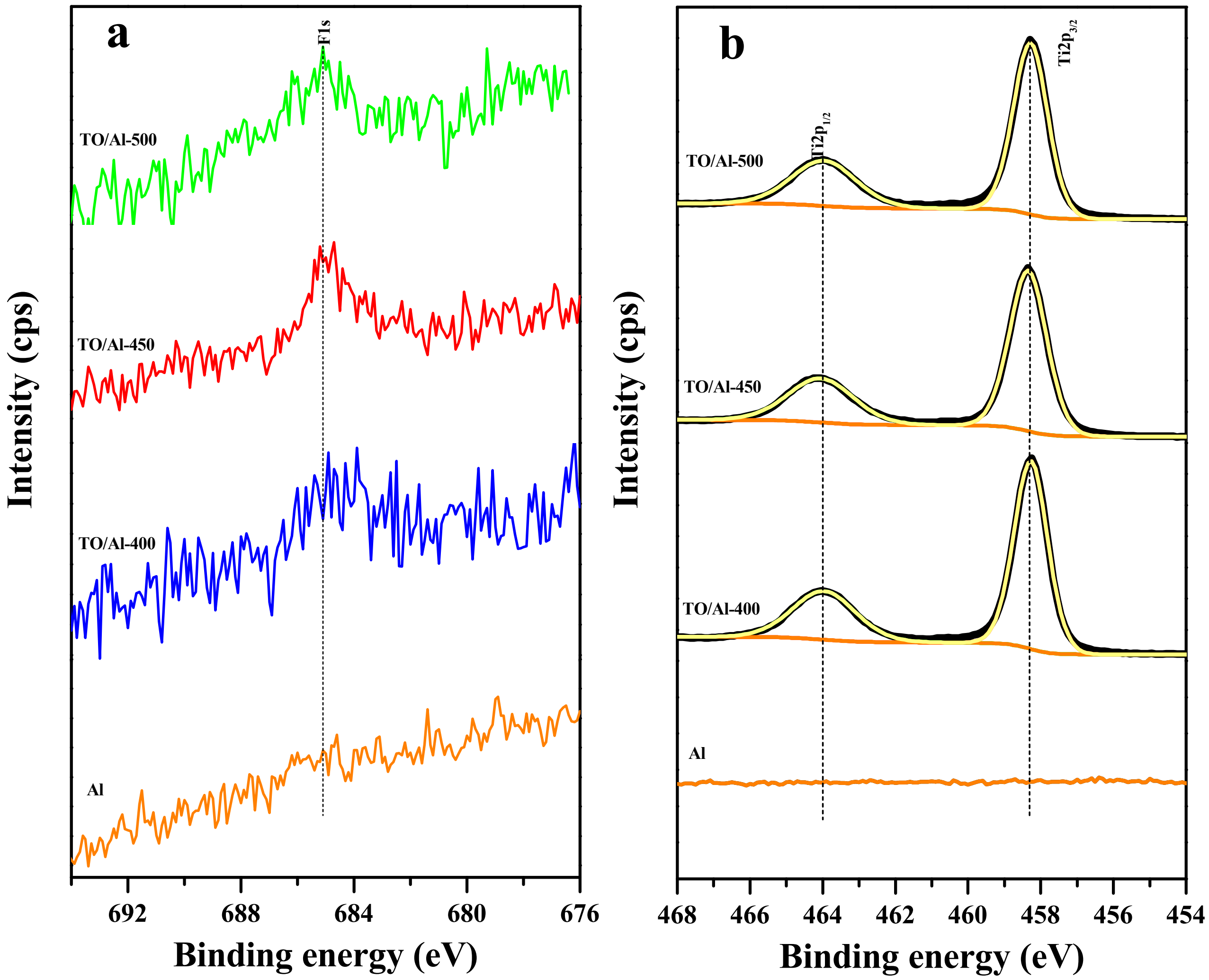

Figure S3. The F1s (**a**) and Ti 2p (**b**) core level of blank Al substrate, TO/Al-400, TO/Al-450, and TO/Al-500.

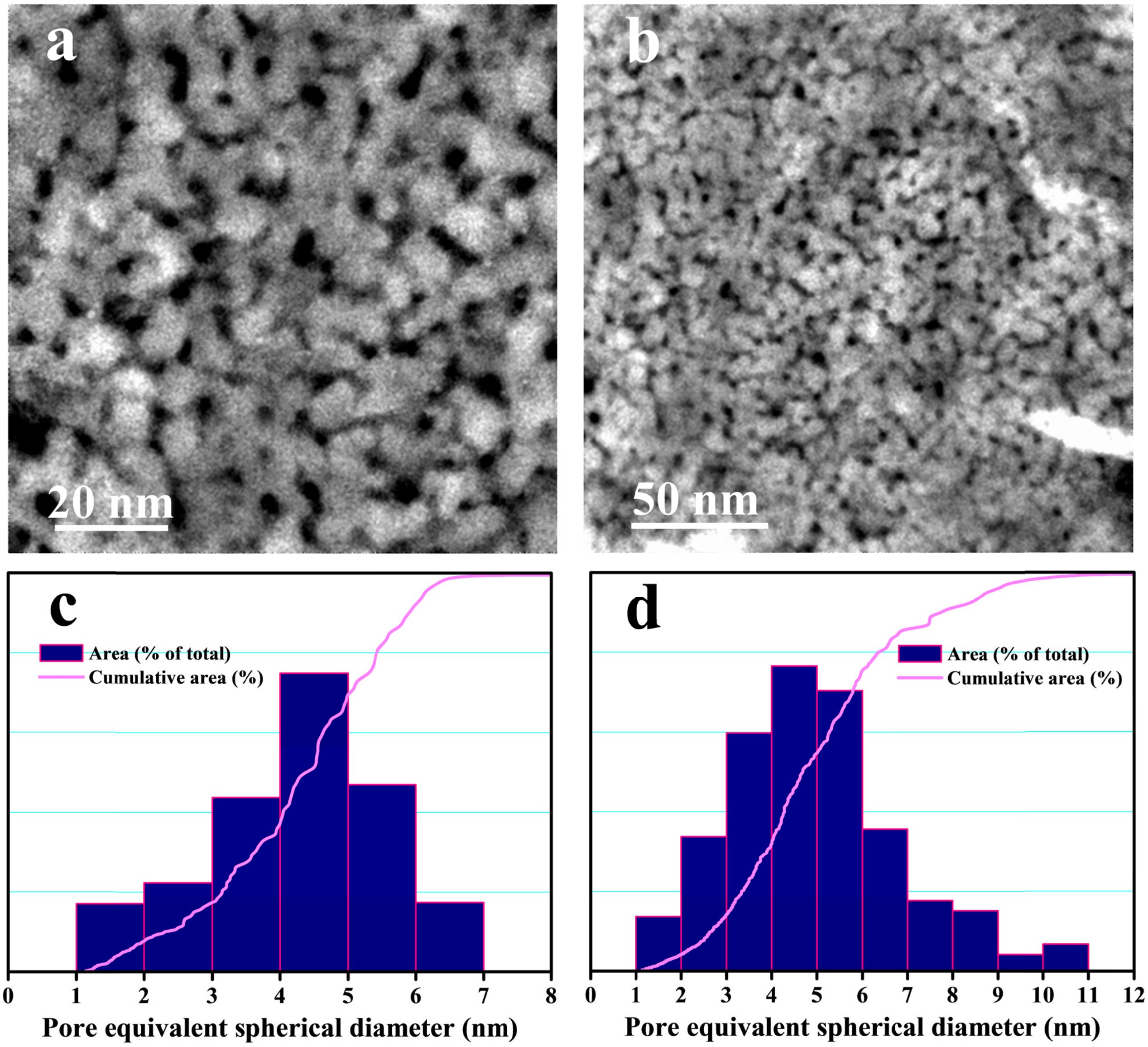

Figure S4. HAADF-STEM images of the mesoporous TiO_2 film and its pore size distribution obtained via ImageJ software. (**a**), in high-magnification; (**b**), in low-magnification; (**c**), pore size distribution corresponding to (**a**); and (**d**), pore size distribution corresponding to (**b**).

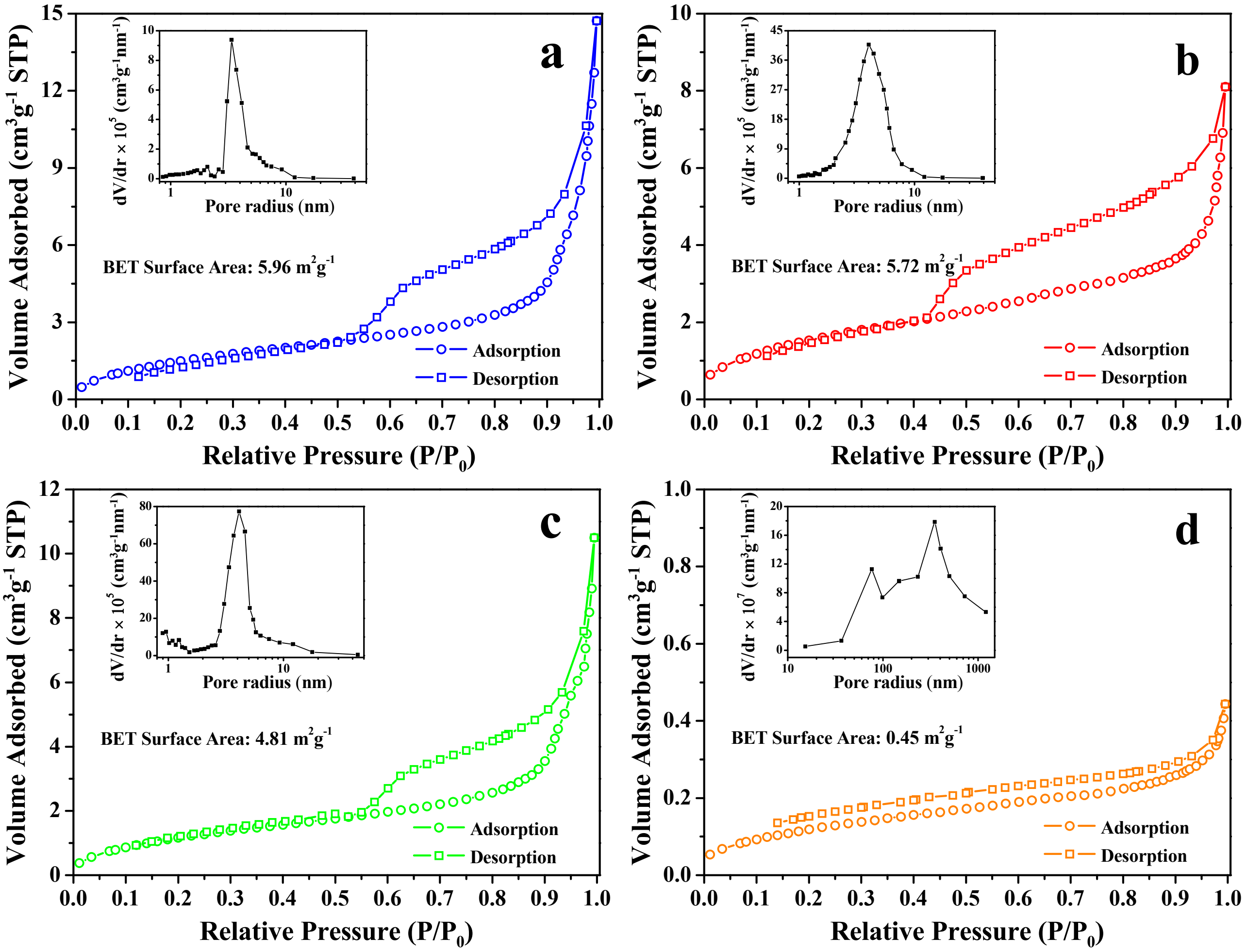

Figure S5. N_2 adsorption–desorption isotherms of TO/Al-400 (**a**), TO/Al-450 (**b**), TO/Al-500 (**c**), and Al (**d**). The insets show the pore-size distribution calculated from the desorption branch.

BET surface area and the pore structure of the samples were characterized via N_2-sorption measurement. All TO/Al samples show similar N_2 adsorption-desorption isotherms with N_2 hysteresis-loop (**Figure S5a-c**), which belong to type IV (BDDT classification), indicating the presence of mesoporous structure. From the pore size distribution (**Figure S5a-c** inset), all TO/Al samples possess mesopores with the size range of 2-10 nm. The surface structure of blank Al substrate is also presented here (**Figure S5d**). Its isotherm plot belongs to type II and its pore size distributes in the range of 40-1000 nm.

Obviously, it is hard to directly measure the surface area of TiO_2 and carbon deposited on the surface of Al substrate. However, its surface area can be estimated by the following equation.

$$m_{Al} \times S_{Al} + \Delta m \times S_{TO+C} = m_{TO/Al} \times S_{TO/Al}$$ **Equation S5**

where S_{Al}, S_{TO+C}, $S_{Al+TO+C}$ are the surface area of blank Al substrate, TiO_2 and carbon deposited on Al substrate, and the whole electrode, respectively. From **Equation S5**, we can calculate that, TiO_2 and carbon on the surface of Al substrate after calcined at 400, 450, and 500 ^{o}C have very high surface areas of 85, 81, and 67 m^2 g^{-1}, respectively.

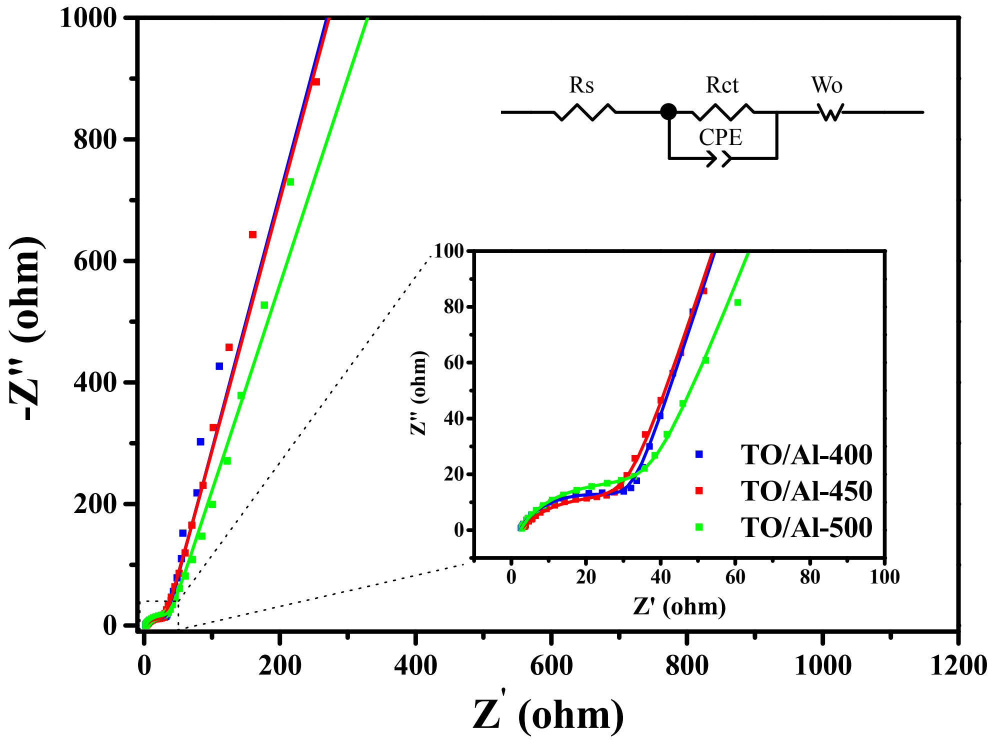

Figure S6. EIS of TO/Al-400, TO/Al-450, and TO/Al-500 at open circuit voltage (~ 2.95 V): the scatter plots show the original data; the line plots show the fitting data. The inset displays the equivalent circuit.

Table S1. The fitted parameters of electrochemical impedance spectra of TO/Al-400, TO/Al-450, and TO/Al-500.

Sample	TO/Al-400 (Error %)	TO/Al-450 (Error %)	TO/Al-500 (Error %)
Rs [Ω]	2.367 (2.596)	2.559 (3.2477)	2.477 (1.8491)
Rct [Ω]	28.79 (2.5714)	28.22 (3.2112)	32.76 (2.4464)
CPE-T [S s^{-n}]	2.7857E-05 (13.434)	8.3176E-05 (14.206)	3.4283E-05 (9.9481)
CPE-P	0.79244 (1.6124)	0.67787 (2.0022)	0.77859 (1.2325)
W_o-R [Ω]	1.319E05 (2.4805)	3.8720E04 (2.224)	4.2465E04 (3.0004)
W_o-T [S s^{-n}]	55.38 (2.9314)	12.59 (3.0839)	13.73 (4.1954)
W_o-P	0.8511 (0.44552)	0.84885 (0.52536)	0.81802 (0.4319)
Chi-Squared	0.0043022	0.0031584	0.0020247

Rs, ohmic resistance in circuit; *Rct*, the charge transfer resistance through the electrode–electrolyte interface; *CPE*, constant phase element; *CPE-T*, constant phase element admittance; *CPE-P*, constant phase element exponent; *Wo*, warburg impendance element; *Wo-R*, warburg impendence element resistance; *Wo-T*, warburg impendence element admittance; *Wo-P*, warburg impendence element exponent.[1]

To get insight into the different electrochemical behaviors of TO/Al electrodes, electrochemical impedance spectra of as-assembled cells were conducted in the frequency range of 100 kHz to 10 mHz, and their Nyquist plots are depicted in **Figure S6**. It can be seen that all plots are composed of a semicircle at high frequencies which are related to the Ohmic resistance and charge transfer resistance, and a short inclined line in low frequency regions which is due to the ion diffusion within the anode.

An equivalent circuit fitting the experimental impedance spectra is proposed to interpret the impedance results, as shown in **Figure S6**. This equivalent circuit model contains *Rs* related to Ohmic resistance in circuit, *Rct* and a constant phase element (CPE) corresponding to the charge transfer resistance through the electrode–electrolyte interface, and a Warburg impedance (Wo) associated with Li^+ diffusion in TiO_2 films. The fitted impedance parameters are listed in **Table S1**. The results of fitting analysis indicate that the *Rs* values of three samples change little with the annealing temperature and are less than 3 Ω, which indicates Al hardly reacts with O_2 from air below 500 ^{o}C (as mentioned above) and keeps high electronic conductivity. However, *Rct* show a little difference after heat-treated at different temperature. The charge transfer resistance value of TO/Al-450 is 28.22 Ω, which is lower than that of TO/Al-400 (28.79 Ω) and TO/Al-500 (32.76 Ω), respectively. As the impedance is in inverse proportional to electrical conductivity, this suggests a higher charge transfer rate for TO/Al-450 electrode, which is mainly caused by the higher content of pyrolytic carbon and better crystallinity in TO/Al-450 as analyzed in main text. It should be pointed out that the *Rct* of three sample are rather low, which favors the high-rate performance.

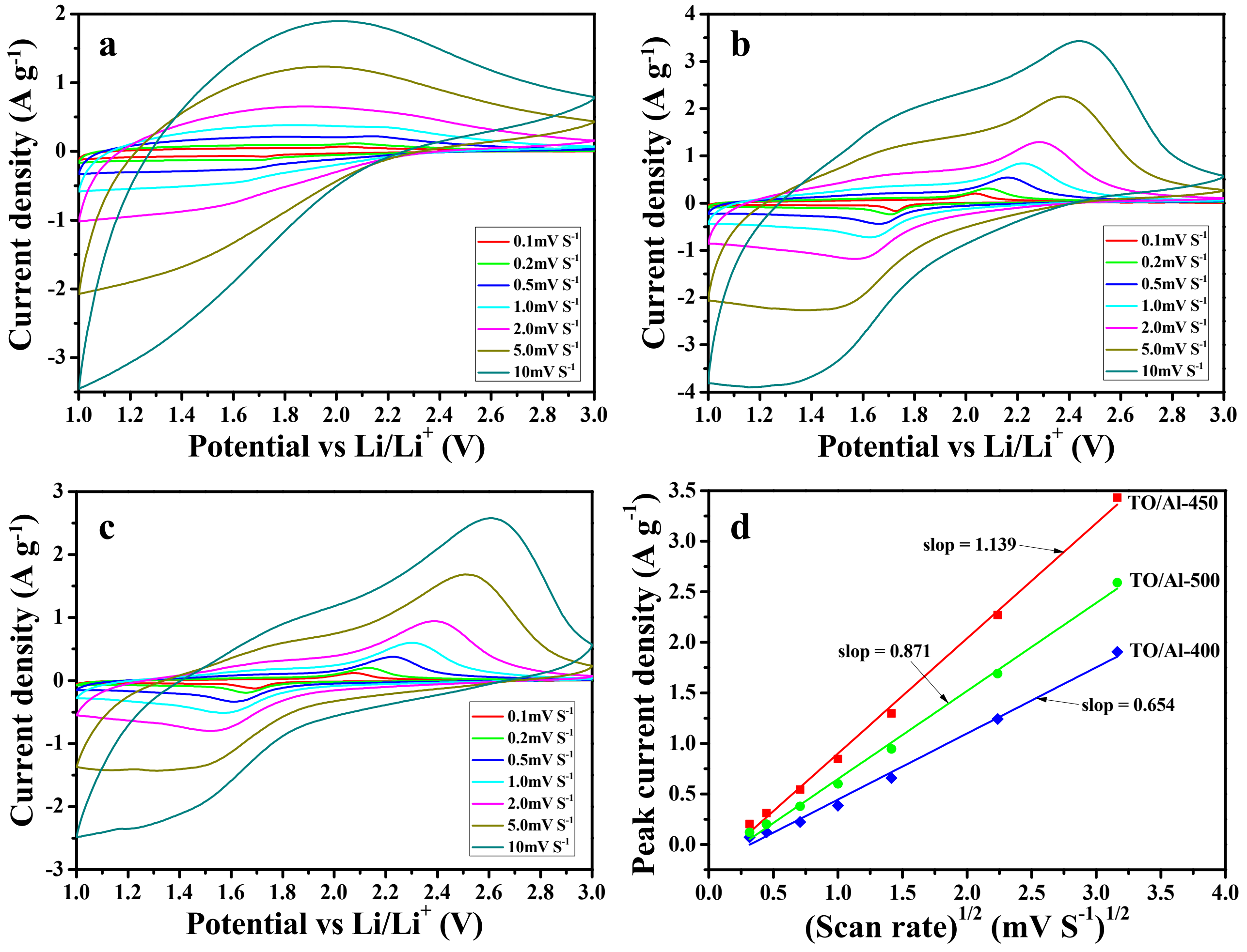

Figure S7. CV curves of TO/Al-400 (**a**) , TO/Al-450 (**b**), TO/Al-500 (**c**) at different scan rates and the linear fitting of peak current density versus square root of the scan rate (**d**): the scatter plots show the original data; the line plots show the fitting data.

Apparent chemical diffusion coefficient of lithium ions in the electrodes, indicating the ionic conductivity, is a characteristic parameter of materials for LIBs, and it can be evaluated via CV measurement. The CV profiles for TO/Al-400, TO/Al-450, and TO/Al-500, obtained through Li-ion insertion/extraction between 1 V and 3 V and under various scan rates, are displayed in **Figure S7**. The prominent reduction and oxidation peaks correspond to the insertion and extraction reactions of lithium ions into and out of TiO_2. To calculate the apparent chemical diffusion coefficient of lithium ions in the three electrodes through the Randles–Sevcik equation (**Equation S6**), the oxidation peak

current versus the square root of the scan rates were redrawn and displayed in **Figure S7d**.

$$I_p = 2.69 \times 10^5 A n^{3/2} C_0 D^{1/2} v^{1/2} \quad \textbf{Equation S6}$$

where I_p, A, n, C_0, D and υ are the peak current density, the electrode electroactive area, the number of electron transferred, the maximum concentration of Li-ions in the electrode (C_0=0.048 mol cm^{-3}),[2,3] the apparent chemical diffusion coefficient, and the scan rate, respectively.

According to the work of Lindstrom and Lindquist,[4,5] at lower scan rates and for thinner films, the electroactive area can be considered as the entire inner area, which was determined by the following equation.[4-6]

$$A_i = \frac{3V_t(1-P)}{r} = \frac{3(V_t - V_p)}{r} \quad \textbf{Equation S7}$$

where A_i is the entire inner area of nanoporous electrode, V_t is the entire film volume, V_p is the pore volume, P is the porosity, and r is the average radius of the active material particles in film. In our case, the scan rate is low (⩽ 10 mV S-1), the film is thin (~ 100 nm) and mesoporous. Thus the inner surface area can be used as electroactive area. From **Figure S7d**, the value of $I_p/υ^{1/2}$ can be calculated. Therefore, according to **Equation S6** and **Equation S7**, the Li^+ apparent chemical diffusion coefficient of TO/Al-400, TO/Al-450, and TO/Al-500 are 1.47×10^{-13}, 4.45×10^{-13}, and 2.61×10^{-13} cm^2 s^{-1}, respectively. It shows that the Li^+ apparent chemical diffusion coefficient increases in order of TO/Al-400 < TO/Al-500 < TO/Al-450.

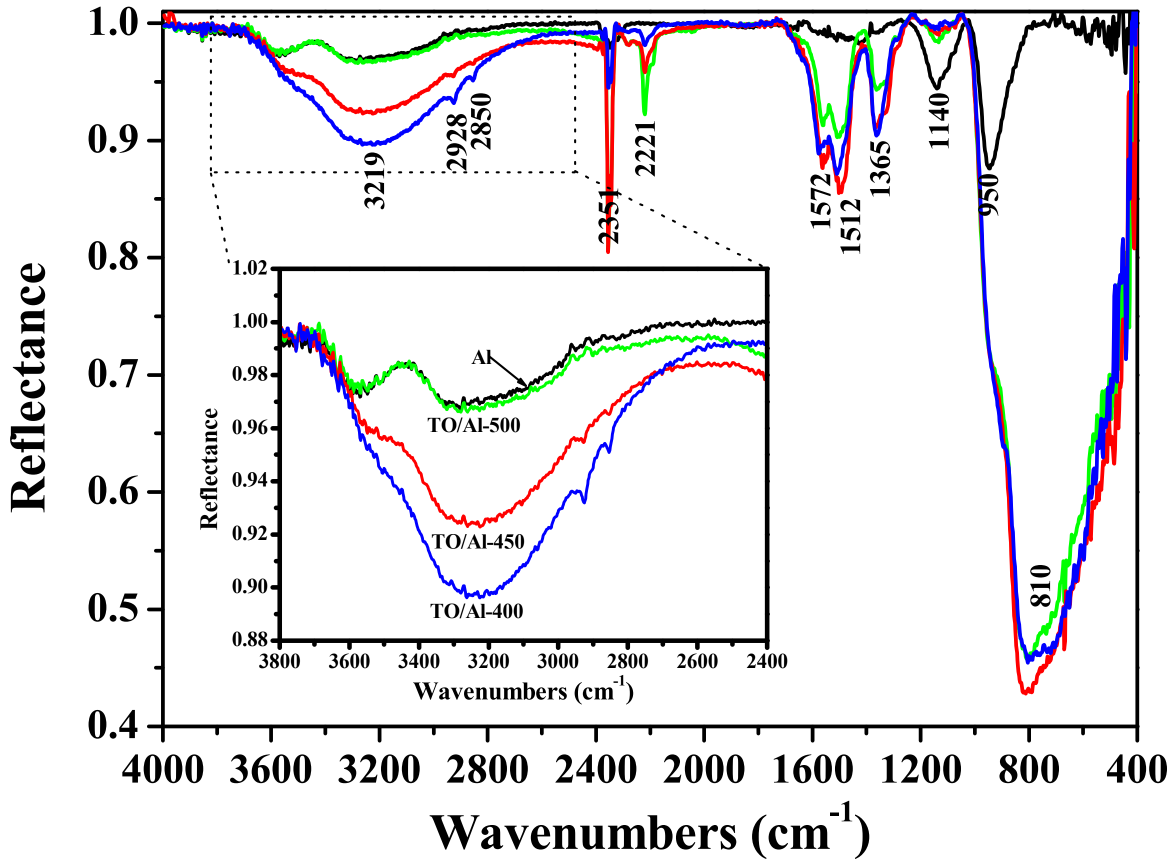

Figure S8. FTIR spectra of Al, TO/Al-400, TO/Al-450 and TO/Al-500.

Table S2. Analysis of reflectance bands appeared in FTIR spectra.

Wavenumbers [cm^{-1}]	Assignment[a]
3219	υ (O-H)
2928	υ_{as} (C-H) in CH_2
2850	υ_s (C-H) in CH_2
2351	υ_{as} (C=O) in CO_2
2221	υ (C≡C) in RC≡CR'
1572	υ (C=C) in RC=CR'
1512	δ_s (C-H) in C=CH
1365	δ_s (C-H) in CH_3
1140	υ_{as} (Al-O) in Al_2O_3
950	υ_s (Al-O) in Al_2O_3
810	υ (Ti-O) in TiO_2

[a] *υ*, stretching mode; *as*, asymmetrical; *s*, symmetrical; *δ*, in-plane bending mode; R, R', alkyl group.

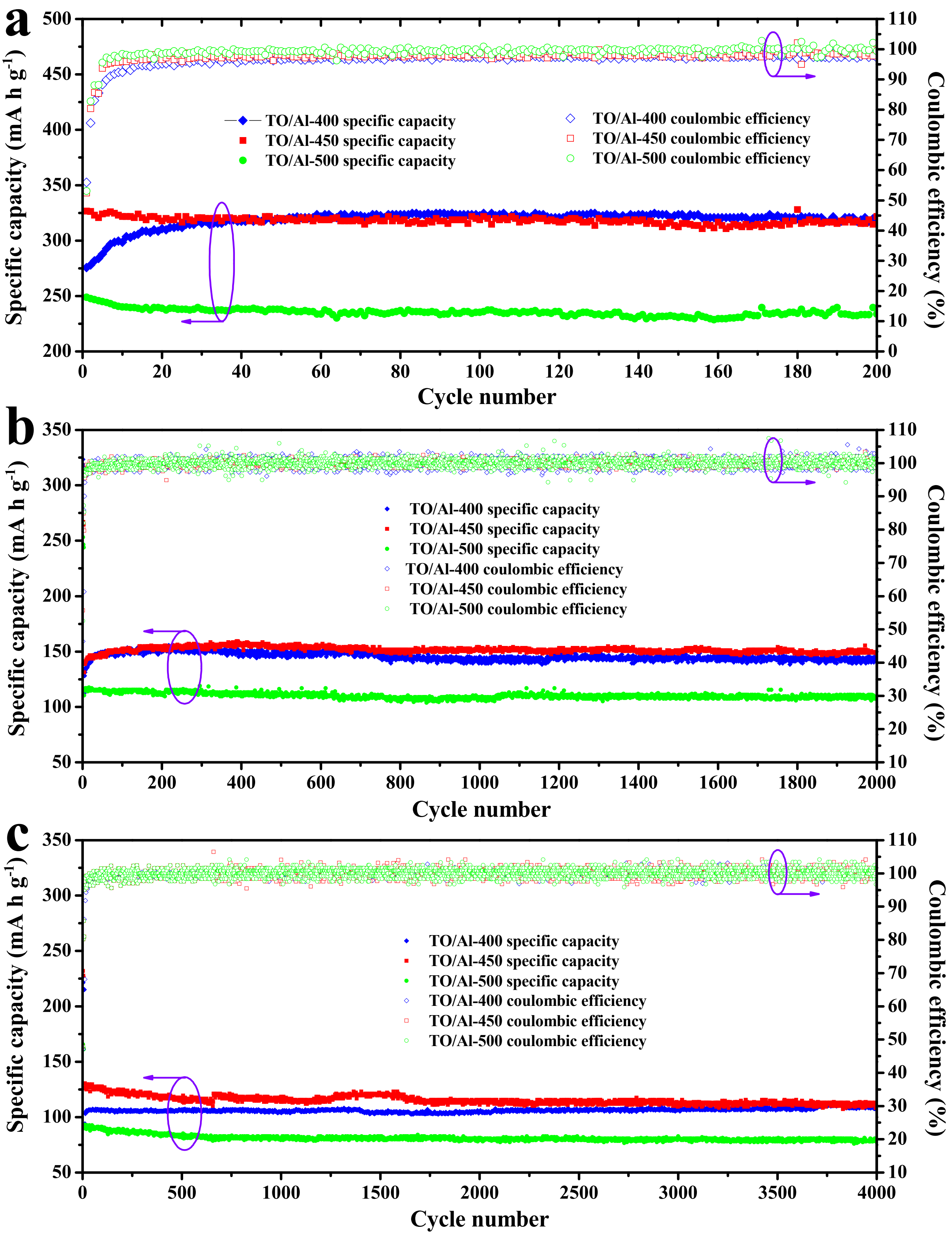

Figure S9. Cycle performance of TO/Al-400, TO/Al-450, and TO/Al-500 in the voltage window of 1-3 V at a current rate of 0.3 C for all cycles (**a**), 0.3 C for the first 3 cycles and 20 C for the next cycles (**b**), 0.3 C for the first 3 cycles and 50 C for the next cycles (**c**), respectively.

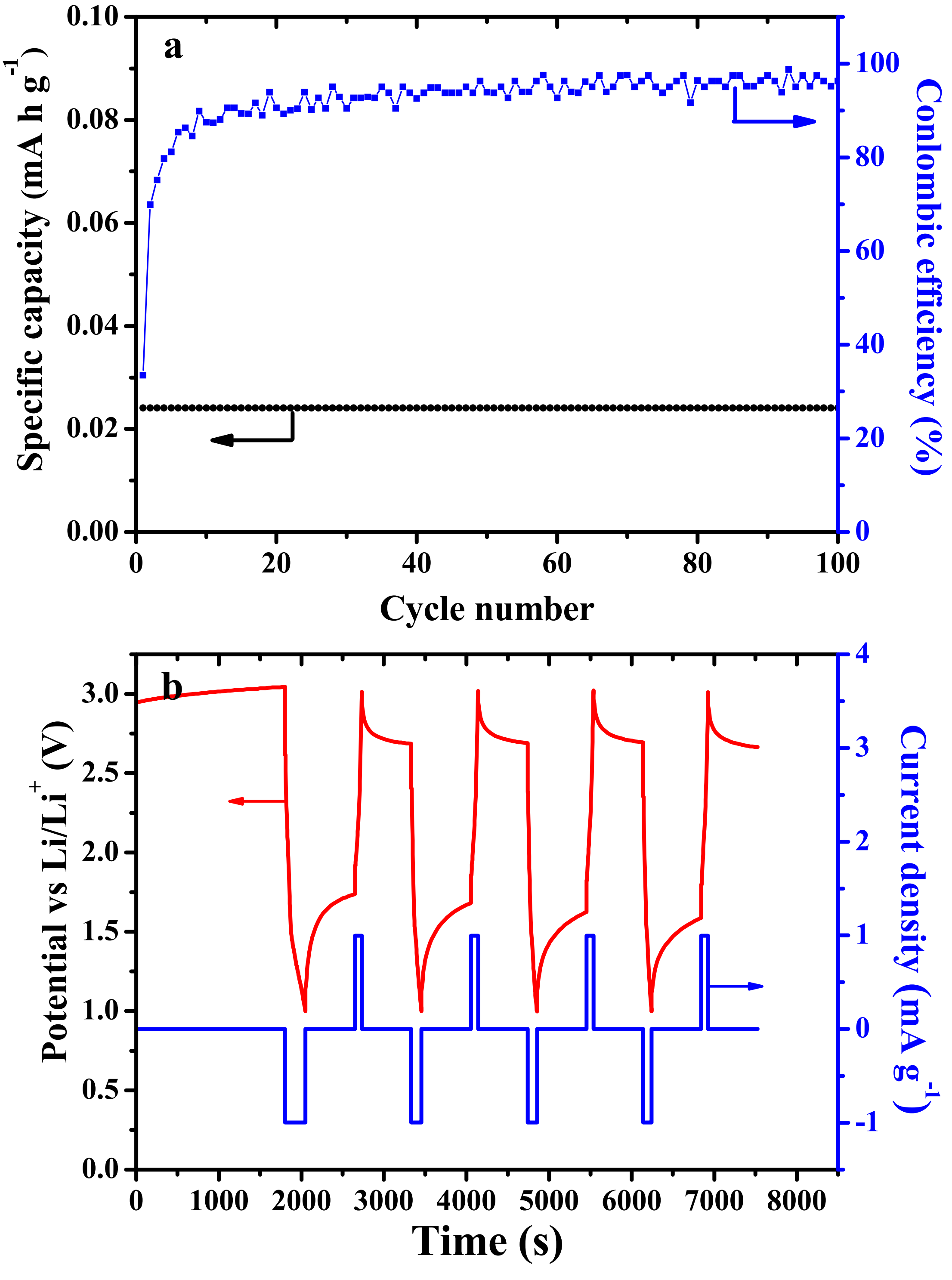

Figure S10. Cycle performance of blank Al substrate at a current of 1 mA g^{-1} within a cut-off voltage window of 1.0-3.0 V (**a**), potential and current density versus time plots of 1st-5th cycle (**b**).

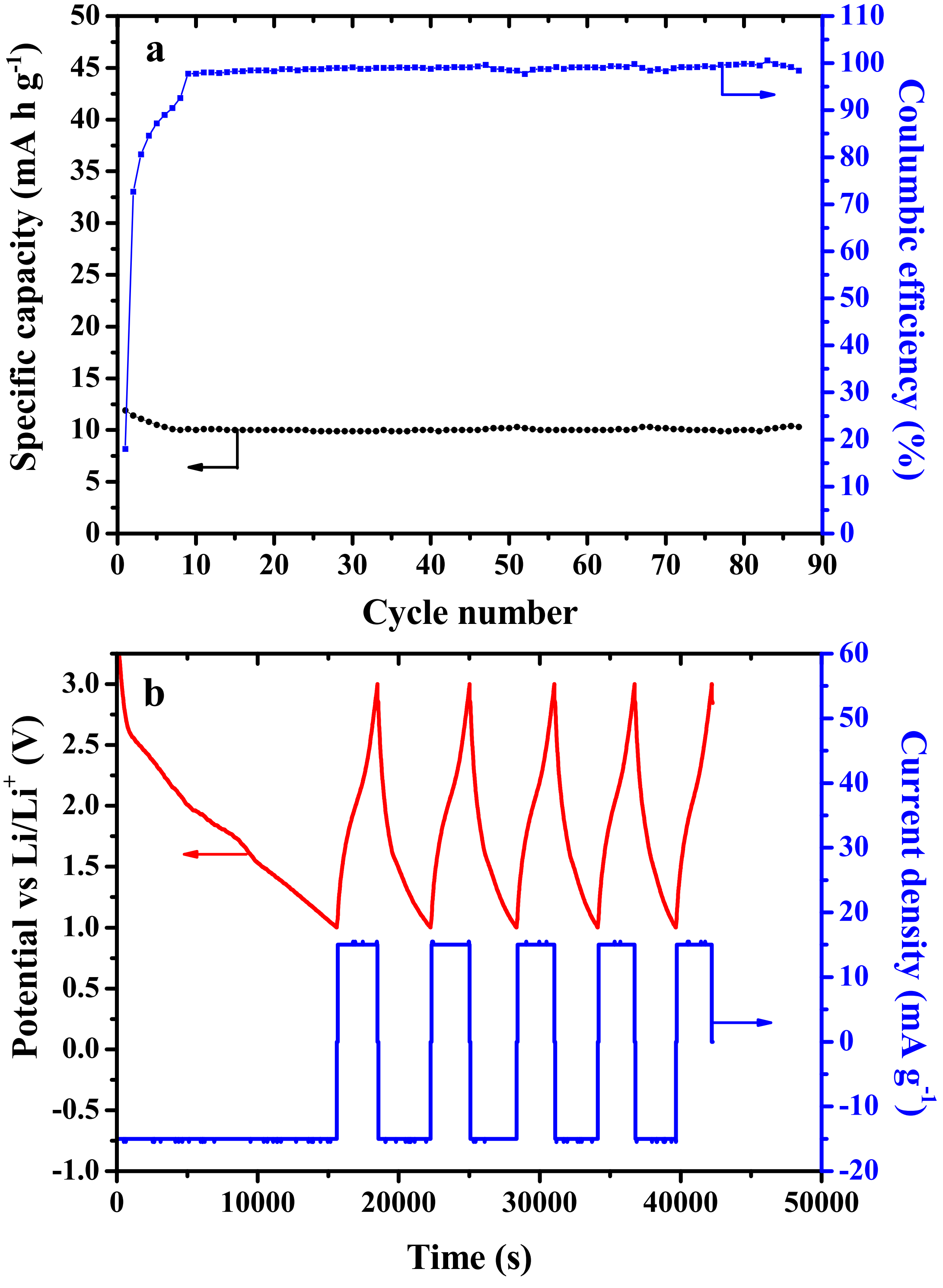

Figure S11. Cycle performance of pyrolytic carbon on Al substrate at a current of 15 mA g^{-1} within a cut-off voltage window of 1.0-3.0 V (**a**), potential and current versus time plots of 1st-5th cycle (**b**).

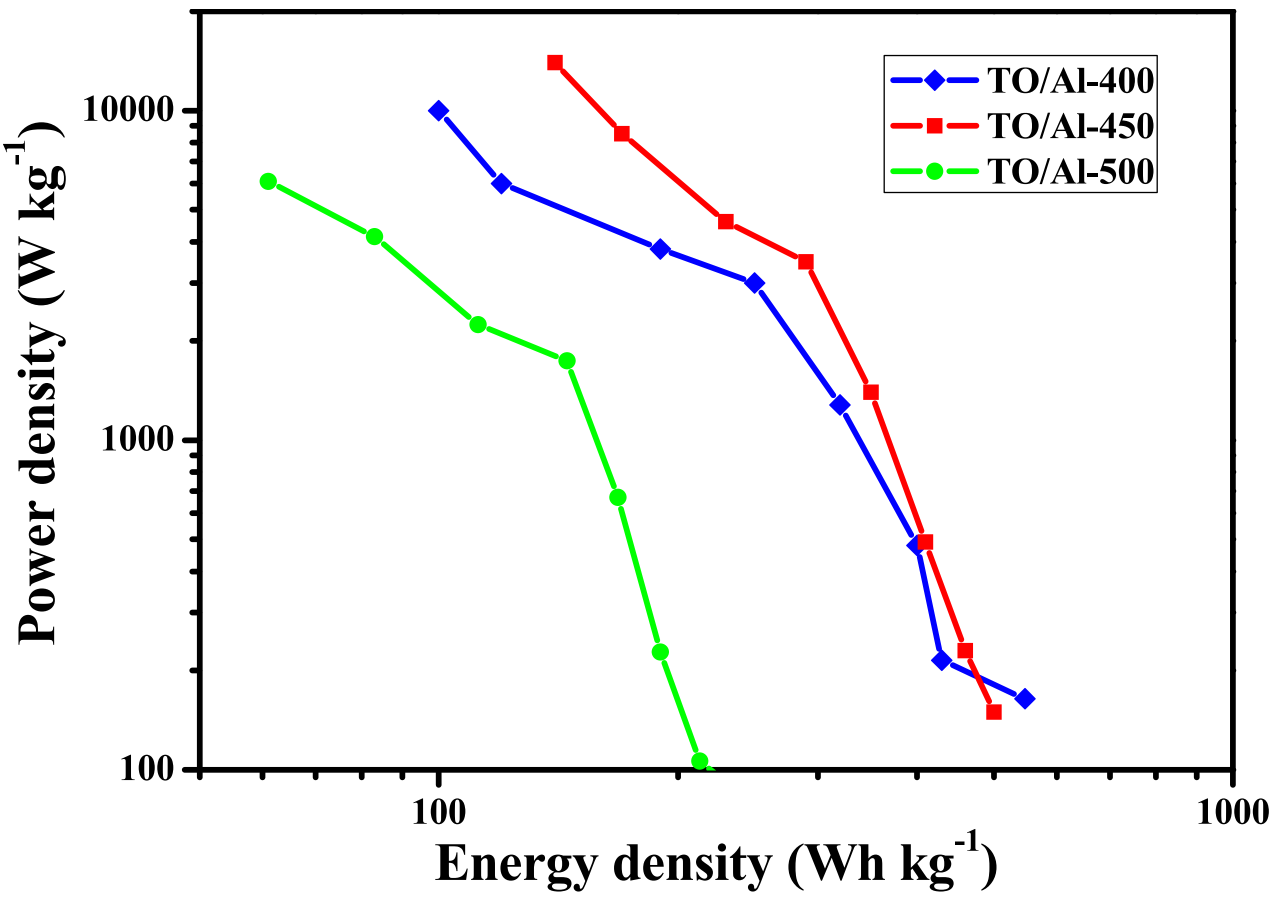

Figure S12. Ragone plots of TO/Al-400, TO/Al-450, and TO/Al-500 in the voltage window of 1.0-3.0 V.

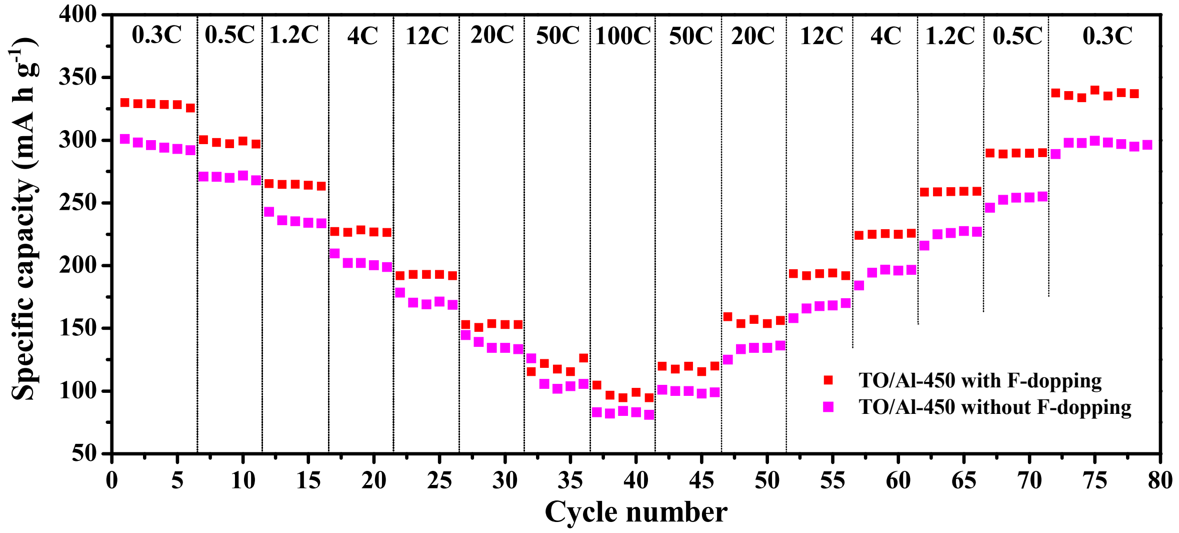

Figure S13. Rate performance of TO/Al-450 with or without F-doping in the voltage window of 1-3 V at a current rate of 0.3 C for the first 3 cycles and 20 C for the next cycles.

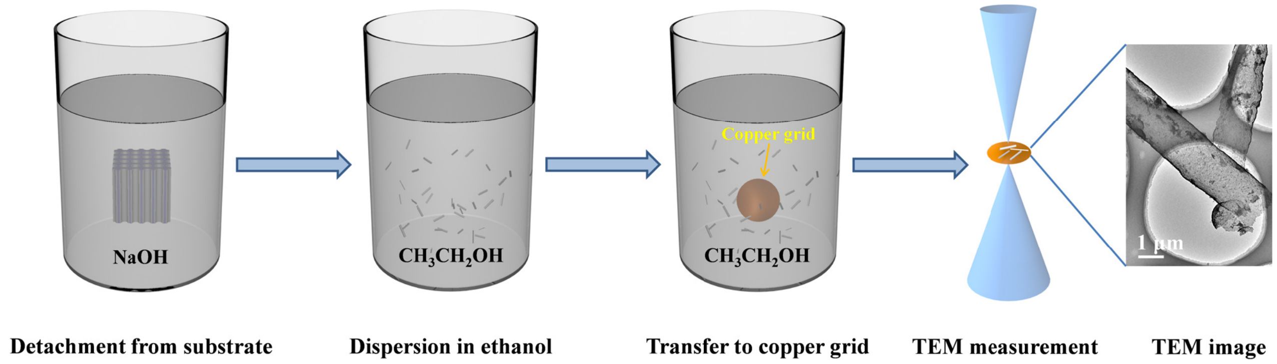

Figure S14. Illustration of typical process used to prepare TEM samples.

References

1 Hosseini, M. G., Sabouri, M. & Shahrabi, T. Comparison of the corrosion protection of mild steel by polypyrrole-phosphate and polypyrrole-tungstenate coatings. *J. Appl. Polym. Sci.* **110**, 2733-2741 (2008).

2 Kavan, L., Rathousky, J., Gratzel, M., Shklover, V. & Zukal, A. Surfactant-templated TiO_2 (anatase): Characteristic features of lithium insertion electrochemistry in organized nanostructures. *J. Phys. Chem. B* **104**, 12012-12020 (2000).

3 Wu, M.-S. *et al.* Electrochemical fabrication of anatase TiO_2 nanostructure as an anode material for aqueous lithium-ion batteries. *J. Power Sources* **185**, 1420-1424 (2008).

4 Lindstrom, H. *et al.* Li^+ ion insertion in TiO_2 (anatase) .1. Chronoamperometry on CVD films and nanoporous films. *J. Phys. Chem. B* **101**, 7710-7716 (1997).

5 Lindström, H. *et al.* Li^+ ion insertion in TiO_2 (Anatase). 2. Voltammetry on nanoporous films. *The Journal of Physical Chemistry B* **101**, 7717-7722 (1997).

6 Kavan, L., Gratzel, M., Rathousky, J. & Zukal, A. Nanocrystalline TiO_2 (anatase) electrodes: Surface morphology, adsorption, and electrochemical properties. *J. Electrochem. Soc.* **143**, 394-400 (1996).
